# Uptake and sorption of aluminium and fluoride by four green algal species

**DOI:** 10.1186/1752-153X-8-8

**Published:** 2014-02-01

**Authors:** Danaé Pitre, Amiel Boullemant, Claude Fortin

**Affiliations:** 1 Institut national de la recherche scientifique, Centre Eau Terre Environnement (INRS-ETE), Université du Québec, 490 rue de la Couronne, G1K 9A9 Québec, QC, Canada; 2 Rio Tinto, Legacy Management, 725 rue Aristide Bergès, Voreppe, Cédex BP 7-38341, France

**Keywords:** Aluminium, Fluoride, Biosorption, Phytoremediation, Algae

## Abstract

**Background:**

We examined the uptake and sorption of aluminium (Al) and fluoride (F) by green algae under conditions similar to those found in the effluents of the aluminium industry. We took into account the speciation of Al in the medium since Al can form stable complexes with F and these complexes may play a role in the uptake and sorption of Al. We compared the capacity of four species of green algae (i.e. *Chlamydomonas reinhardtii*, *Pseudokirchneriella subcapitata*, *Chlorella vulgaris,* and *Scenedesmus obliquus*) to accumulate and adsorb Al and F. The selected algae were exposed during 4 days, covering all growth phases of algae, to a synthetic medium containing Al and F at pH 7.0. During this period, dissolved Al as well as cellular growth were followed closely. At the end of the exposure period, the solutions were filtered in order to harvest the algal cells. The cells were then rinsed with enough ethylene diaminetetraacetic acid to remove loosely bound ions from the algal surface, determined from the filtrates. Finally, the filters were digested in order to quantify cellular uptake.

**Results:**

Little difference in Al removal was observed between species. Aluminium sorption (15%) and uptake (26%) were highest in *P. subcapitata*, followed by *C. reinhardtii* (7% and 17% respectively), *S. obliquus* (13% and 5%), and *C. vulgaris* (7% and 2%). However, none of these species showed significant uptake or sorption of F. We also studied the influence of pH on the uptake and sorption of Al and F by *P. subcapitata.* We measured a combined uptake and sorption of Al of 50% at pH 7.5, of 41% at pH 7.0, and of 4% at pH 5.5. Thus, accumulation was reduced with acidification of the medium as expected by the increased competition with protons and possibly by a reduced bioavailability of the Al-F complexes which dominated the solution at low pH.

**Conclusion:**

Out of the four tested species, *P. subcapitata* showed the highest sorption of aluminium and fluoride under our test conditions. These results provide key information on the development of an environmental biotechnology which can be applied to industrial effluents.

## Background

Canada is the third largest world producer of aluminium with over two million tons per year [1]. Aluminium production thus represents a significant source of Al and F inputs to aquatic environments. In order to reduce aluminium concentrations in effluents, conventional methods such as ion exchange resins or chemical precipitation can be used, but these are costly and may be inefficient when metal concentrations are low [2]. Biological techniques can thus be considered as a complement to proactively reduce the concentrations of several elements of environmental concern.

Cellular membranes contain structures, such as proteins, which allow the entry of ions. Moreover, these structures as well as cell walls have functional groups that may bind ions [2-5]. Algae may bind metals through a sorption process which is rapid and reversible [6,7]. This process is coupled with uptake which is a slow, irreversible process where the metal is internalized by the cell [8-10].

Based on several previous studies that have shown that algae can efficiently remove metals from industrial effluents [2,11,12], we decided to investigate if this was applicable to Al and F. Precisely, we examined sorption and uptake of Al and F by four species of green algae. Since Al can form complexes with different ligands such as F, we took into account the chemical speciation of the metal in solution and investigated the influence of Al-F complexes on the sorption and uptake of Al and F. The formation of such metallic complexes depends on the availability of the metal in solution, the pH, the ionic strength of the medium, and the concentration of complexing ligands [13]. Finally, we studied the influence of pH on the sorption and uptake of F and Al by one selected species of algae.

This study provides essential data that will contribute to determine the feasibility of using green algae to improve the wastewater treatment for Al and F removal from the effluents of aluminium smelters.

## Results and discussion

### Algal growth

Growth controls showed that *C. vulgaris* was the species with the greatest cell abundance after 96 h in the selected culture medium (2.4 ± 0.3 × 10^6^ cell ml^-1^). However, it was also the only species for which cellular growth was inhibited when Al was present in the medium along with F, suggesting a toxic effect although this was only observed in one of the two replicates. Similar results have been observed with the genus *Chlorella*[14]: the growth of *C. pyrenoidosa* was also inhibited by the presence of Al (1.6 μM) and F (5 μM) at pH 5.5. Therefore, *C. vulgaris* may perhaps be more sensitive to Al and F and might not represent a good candidate for the treatment of aluminium smelter effluents.

### Fluoride accumulation

Concentrations of adsorbed and cellular F were too low to be measured with the fluoride ion selective electrode. Also, the measured initial and final dissolved F (Table 1) did not indicate any noticeable decrease in fluoride. We anticipated that F^-^ would not sorb notably to algal cells since the membrane is negatively charged at pH 7.0. However, we also hypothesized that F could be sorbed as Al-F complexes. At pH 7.0, Al-F complexes were present but at very low concentrations (AlF_4_^-^, AlF_3_ (aq), AlOHF_2_ (aq) and AlF_2_^+^). If these complexes were sorbed at all, the resulting [F]_sorbed_ was too low and could not be detected by the electrode which had a quantification limit of 2.6 μM.

**Table 1:** **Initial (t = 0) and final (t = 96 h) concentrations of fluoride in solution for experiments with each species of green algae (pH = 7.0; detection limit of the selective electrode: 0.02 mg L**^**-1**^**(1 μM); quantification limit of the selective electrode: 0.05 mg L**^**-1**^**(2.6 μM))**

**Treatment F**	**Initial [F] (mg L**^**-1**^**)**	**Final [F] (mg L**^**-1**^**)**
***C. reinhardtii***	7.2 ± 0.2	6.7 ± 0.2
***P. subcapitata***	6.95 ± 0.02	7.1 ± 0.3
***C. vulgaris***	7.3 ± 0.2	7.36 ± 0.03
***S. obliquus***	7.4 ± 0.1	7.7 ± 0.1
**Treatment Al + F**	**Initial [F] (mg L**^**-1**^**)**	**Final [F] (mg L**^**-1**^**)**
***C. reinhardtii***	7.1 ± 0.3	6.6 ± 0.2
***P. subcapitata***	7.2 ± 0.2	7.2 ± 0.3
***C. vulgaris***	7.1 ± 0.3	7.3 ± 0.1
***S. obliquus***	7.4 ± 0.3	7.9 ± 0.4

Uptake of F was also negligible. It has been suggested that the transport of F through the membrane would result primarily from the non-ionic diffusion of HF [15]. Moreover, even though the calculated concentration of HF was very low at pH 7.0 in both treatments (~0.05 μM), this species constantly regenerates itself in solution in order to preserve the equilibrium between species and the concentration of HF cannot be considered as a limiting factor of uptake. Therefore, we cannot confirm that HF or any other species could diffuse through the membrane under our test conditions. Similarly, since there was no significant accumulation of F in either treatment, we could not determine whether Al had an effect on the accumulation of F at pH 7.0 under our test conditions.

### Aluminium accumulation

**Figure 1:**
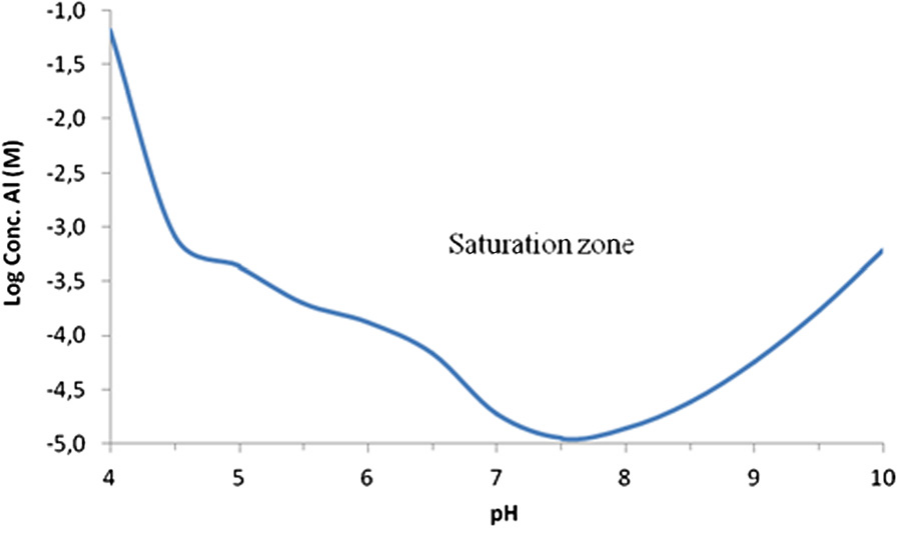
**Aluminium solubility as a function of pH.** Concentration of aluminium used for the exposures was of the order of 10^-5^ M ([Al_T_] = 10.4 μM, [F_T_] = 379 μM, Table 6).

We tracked the dissolved Al regularly during the exposure period and observed a rapid decrease within the first hour (Table 2). Mass balance calculations using the values of dissolved, sorbed, cellular, and particulate Al suggest that this decrease was mostly due to precipitation but also due to adsorption to the cell surface, (Table 3). Afterwards, Al likely was removed at a slower rate due to cellular uptake. The average recovery of Al was 85% with a range of 64 to 106%. The presence of a particulate phase in our growth media was unexpected based on thermodynamic calculations which showed that the solutions were undersaturated with respect to the microcrystalline gibbsite (Al(OH)_3_(s); Figure 1). Our control flasks without algae also showed significant decreases in dissolved Al over time (initial [Al]_meas._ = 235–265 μg L^-1^; final [Al]_meas._ = 41–212 μg L^-1^), indicating that our solutions were indeed oversaturated with an Al mineral phase.

**Table 2:** **Variation of dissolved aluminium in solution (μg L**^**-1**^**) during the experiments (detection limit of the ICP-AES: 1 μg L**^**-1**^**(37 nM); quantification limit of the ICP-AES: 5 μg L**^**-1**^**(185 nM))**

	***C. reinhardtii***	***P. subcapitata***	***C. vulgaris***	***S. obliquus***
**t = 0 h**	251 ± 7	240 ± 31	262 ± 3	240 ± 2
**t = 1 h**	63 ± 17	68 ± 18	151 ± 10	93 ± 5
**t = 6 h**	61 ± 16	65 ± 19	144 ± 9	80 ± 1
**t = 24 h**	59 ± 15	60 ± 17	134 ± 12	82 ± 15
**t = 48 h**	60 ± 13	56 ± 16	126 ± 9	75 ± 8
**t = 72 h**	59 ± 11	54 ± 17	124 ± 9	72 ± 8
**t = 96 h**	56 ± 6	51 ± 20	124 ± 10	82 ± 32

**Table 3:** **Mass balances (μg) for aluminium in the experiments containing both Al and F ([Al**_**T**_**] = 10.4 μM; 281 μg L**^**-1**^**, [F**_**T**_**] = 379 μM; 7.2 mg L**^**-1**^**, pH = 7.0)**

**Experiments**	**Dissolved Al**	**Dissolved Al**	**Sorbed Al**	**Cellular Al**	**Particulate Al**	**Recovery (%)**
	**t = 0 h**	**t = 96 h**			**t = 96 h**	
*C. reinhardtii*–A	256 ± 6	58 ± 8	19 ± 2	35 ± 10	109 ± 16	87 ± 7
*C. reinhardtii*–B	247 ± 6	53 ± 2	16 ± 4	52 ± 9	53 ± 10	71 ± 4
*P. subcapitata*–A	257 ± 6	35 ± 1	24 ± 5	74 ± 19	31 ± 8	64 ± 6
*P. subcapitata*–B	263 ± 6	77 ± 2	54 ± 13	63 ± 21	26 ± 10	83.4 ± 0.3
*C. vulgaris*–A	262 ± 3	124 ± 10	22 ± 2	11 ± 2	71 ± 4	87 ± 2
*C. vulgaris*–B	251 ± 2	251 ± 1	11.8 ± 0.3	0.2 ± 0.2	4 ± 9	106 ± 3
*S. obliquus*–A	240 ± 2	62 ± 4	22 ± 3	9 ± 1	133 ± 7	94 ± 2
*S. obliquus*–B	239 ± 2	101 ± 37	42 ± 8	14 ± 1	74.4 ± 0.3	86 ± 2

Even though Al did not contribute to the accumulation of F, we can conclude that F does contribute to the accumulation of Al, not as a result of the accumulation of Al-F complexes, but simply by buffering Al concentrations and thus limiting its precipitation. In fact, with no F in solution, based on thermodynamic calculations, Al would have been expected to precipitate at the concentration used. The presence of F did contribute to an increase in the solubility of Al, but our media were in fact very close to the saturation zone at pH 7.0 (Figure 1). Precipitation did vary from one experiment to another and may have limited the accumulation process by decreasing Al bioavailability, but we believe this is still representative of typical industrial effluents where oversaturation is frequent.

Regarding the accumulation capacity of each species, *C. vulgaris* showed a significantly lower accumulation of Al (35 fg cell^-1^, Tukey, P = 0.03) while *S. obliquus, P. subcapitata* and *C. reinhardtii* showed similar accumulations (83 fg cell^-1^, Tukey, P = 1.00) (Table 4). However, since *P. subcapitata* had higher cell densities (1.9 ± 0.1 × 10^6^ cell mL^-1^) after 96 h compared to the other species tested (7.5 ± 0.7 × 10^5^ cell mL^-1^ for *C. reinhardtii*; 9.6 ± 1.5 × 10^5^ cell mL^-1^ for *C. vulgaris*; 4.7 ± 0.9 × 10^5^ cell mL^-1^ for *S. obliquus*), the relative accumulation of Al by this species (41%) was significantly higher than accumulation by the other species (9%, 18%, 25%) (Tukey, P < 0.01). If we consider only the sorption process, even though *S. obliquus* showed the highest absolute sorption (60 fg cell^-1^, Tukey, P < 0.01) of the species tested, its relative sorption remains comparable to the sorption by *P. subcapitata* (respectively 13% and 15%, Tukey, P = 0.93). According to these results, *P. subcapitata* could be used alone or combined with *S. obliquus* for an eventual treatment of effluents, assuming such treatment occurs over a similar time frame. For treatments over a shorter period of time, *S. obliquus* might be a good candidate.

**Table 4:** **Aluminium absolute (in fg cell**^**-1**^**) and relative (% of total Al present in solution) accumulation by the four species of green algae tested ([Al**_**T**_**] = 10.4 μM; 281 μg L**^**-1**^**, [F**_**T**_**] = 379 μM; 7.2 mg L**^**-1**^**, pH = 7.0)**

		**Sorption**	**Uptake**	**Total accumulation**
***C. reinhardtii***	**fg cell**^**-1**^	**24 ± 5**	**59 ± 16**	**83 ± 14**
	%	7 ± 1	17 ± 5	25 ± 4
***P. subcapitata***	**fg cell**^**-1**^	**27 ± 7**	**56 ± 32**	**83 ± 31**
	%	15 ± 7	26 ± 7	41 ± 5
***C. vulgaris***	**fg cell**^**-1**^	**23 ± 5**	11 ± 4*	35 ± 8*
	%	7 ± 2	2 ± 2	9 ± 4
***S. obliquus***	**fg cell**^**-1**^	60 ± 17*	**22 ± 4**	**83 ± 20**
	%	13 ± 5	5 ± 1	18 ± 6

*P < 0.05.

### Influence of pH on fluoride accumulation

**Figure 2:**
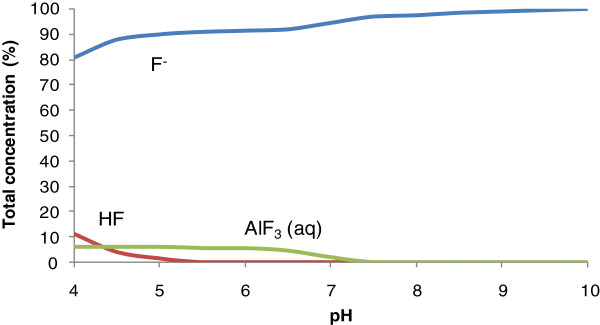
**Calculated fluoride speciation as a function of pH in the presence of aluminium ([Al] = 10.4 μM, [F] = 379 μM, Table**6**).**

Accumulation of F remained negligible at all of the pH values tested. Sorption of F would have been more probable in conditions below the isoelectric point when the membrane becomes positively charged, but at pH 5.5, the membrane remains most likely negatively charged [9]. As mentioned previously, F uptake may perhaps result from non-ionic diffusion of HF through the membrane [15]. However, the relative presence of this form remains negligible, even at pH 5.5. Within our treatment conditions, the proportion of HF species becomes substantial only below pH 5.0 (Figure 2). Fluoride accumulation by the green algal species tested is therefore not efficient enough to be considered as a tool for F removal from effluents. Based on previous studies, we can assume that F accumulation by algae is possible [15-17]. However, it remains unclear which conditions are required to improve F accumulation by algae and if algal cells need to undergo a pre-treatment to be efficient at removing F.

### Influence of pH on aluminium accumulation

As anticipated, accumulation of Al was influenced by pH (Table 5). Accumulation was significantly lower at pH 5.5 (4%, Tukey, P < 0.01) while it was similar at pH 7.0 and 7.5 (respectively 41% and 50%, Tukey, P > 0.05). However, surface sorption was significantly higher at pH 7.5 compared to pH 7.0 (respectively 28% and 15%, Tukey, P = 0.02). A lower pH leads to an increase in dissolved Al and in the proportion of free ions (Al^3+^). However, along with an increasing proportion of free metal ions, acidification leads to a decreasing number of available binding sites on the membrane following protonation [18]. Therefore, lower uptake at pH 5.5 could be explained by an increased competition between protons and free metal ions for the same binding sites, as expected based on the Biotic Ligand Model [10].

**Table 5:** **Effect of pH on aluminium absolute (in fg cell**^**-1**^**) and relative (% of total Al present in solution) accumulation by** ***P. subcapitata*** **([Al**_**T**_**] = 10.4 μM; 281 μg L**^**-1**^**, [F**_**T**_**] = 379 μM; 7.2 mg L**^**-1**^**, pH = 7.0)**

		**Sorption**	**Uptake**	**Total accumulation**
**pH 5.5**	**fg cell**^**-1**^	14 ± 2*	1 ± 1*	15 ± 2*
	%	3.9 ± 0.5	0.3 ± 0.4	4.2 ± 0.8
**pH 7.0**	**fg cell**^**-1**^	27 ± 6*	**56 ± 17**	**83 ± 13**
	%	15 ± 7	26 ± 7	41 ± 5
**pH 7.5**	**fg cell**^**-1**^	43 ± 5*	**34 ± 5**	**77 ± 1**
	%	28 ± 1	22 ± 5	50 ± 4

*P < 0.05.

**Figure 3:**
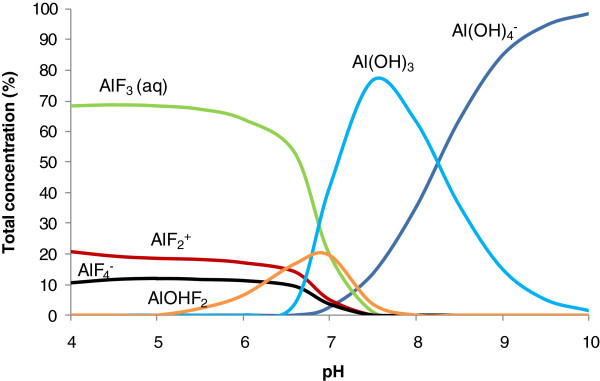
**Calculated aluminium speciation as a function of pH in the presence of fluoride ([Al**_**T**_**] = 10.4 μM, [F**_**T**_**] = 379 μM, Table**6**).**

Differences in uptake could also be explained by the nature of complexes present in solution. Binary fluoro-complexes of aluminium dominate the solution at pH 5.5 while hydroxo-complexes dominate at pH 7.0 and pH 7.5 (Figure 3). Based on the residual charge of metallic species present in solution, we can hypothesize that the complex AlF_2_^+^ would have more affinity for the negatively charged membrane, at any tested pH, than the other neutral or negatively charged complexes (Figure 3). Since this species was more abundant at pH 5.5 than at pH 7.0, the total Al accumulation should also have been more important at pH 5.5. Along with the increased abundance of free ions (Al^3+^) at pH 5.5, we can conclude that, given our test conditions, the competition by protons is a more important factor for Al accumulation than the nature of species in solution.

Overall, whether Al enters the cell solely as free ions or also as Al-F or Al-OH complexes remains unclear. In fact, no studies yet have been able to clearly identify uptake mechanisms for Al [9]. As these experiments were conducted over long exposure periods (4 days), we must also consider that equilibrium may have been disturbed over time with the probable exudation of biogenic ligands that may have played a role in the speciation of Al.

### Experimental

For this study, four species of green algae were selected: *Chlamydomonas reinhardtii* (Canadian Phycological Culture Center, CPCC 11), *Pseudokirchneriella subcapitata* (CPCC 37), *Chlorella vulgaris* (CPCC 90), and *Scenedesmus obliquus* (CPCC 5). These species were selected for their ease of growth and the availability of data on metal uptake/adsorption in the literature. Batch cultures were maintained in acid-washed 250 mL polycarbonate Erlenmeyer flasks containing 100 mL of sterile Modified High Salt Medium (MHSM-1; ionic composition is presented in Table 6[19]). This same medium was used to grow the algae and to expose them to aluminium and fluoride. However, since the addition of fluoride in solution modifies the ionic strength of the medium, the molar concentration of KNO_3_ was adjusted consequently when fluoride was added in order to maintain a constant ionic strength throughout our experiments. Cultures were maintained at pH 7.0, at a temperature of 20.0°C ± 0.1°C, with rotary agitation (60 rpm) and under constant illumination (Cool White Fluorescent Tubes, 100 ± 10 μE∙m^‒2^∙s^‒1^). Every week, 2 mL of the cultures were transferred to a fresh medium in order to maintain healthy cell cultures.

**Table 6:** Ionic composition (M) of the culture media used

**Ions**	**MHSM-1**^**a**^	**MHSM-1 + F**
**NH**_**4**_	9.37 × 10^-4^	9.37 × 10^-4^
**Cl**	5.98 × 10^-6^	5.98 × 10^-6^
**F**	-	3.79 × 10^-4^
**K**	4.22 × 10^-3^	3.84 × 10^-3^
**PO**_**4**_	1.37 × 10^-4^	1.37 × 10^-4^
**CO**_**3**_	atm.^b^	atm.^b^
**NO**_**3**_	5.07 × 10^-3^	4.69 × 10^-3^
**SO**_**4**_	8.12 × 10^-5^	8.12 × 10^-5^
**Mg**	8.12 × 10^-5^	8.12 × 10^-5^
**Ca**	6.80 × 10^-5^	6.80 × 10^-5^
**Na**	1.02 × 10^-4^	4.81 × 10^-4^
**BO**_**3**_	3.01 × 10^-6^	3.01 × 10^-6^
**Mn**	2.10 × 10^-6^	2.10 × 10^-6^
**EDTA**	8.06 × 10^-7^	8.06 × 10^-7^
**Fe**	5.92 × 10^-7^	5.92 × 10^-7^
**MoO**_**4**_	3.00 × 10^-8^	3.00 × 10^-8^
**Zn**	2.43 × 10^-8^	2.43 × 10^-8^
**Co**	1.09 × 10^-8^	1.09 × 10^-8^
**Cu**	7.04 × 10^-11^	7.04 × 10^-11^

a: See [19].

b: No carbonates were added since their concentration is adjusted with the atmosphere by gaseous exchange.

Cells were harvested in their exponential growth phase by centrifugation, rinsed and re-suspended in fresh growth medium to a concentration of 20,000 cell mL^-1^. Experiments with all four species were conducted in the same growth medium at pH 7.0. Algae were exposed simultaneously to F and Al and, in order to study the role of Al-F complexes of Al on the accumulation of F, they were also exposed to F only. Both treatments were repeated in triplicates. We used constant total concentrations of F (379 μM; 7.2 mg L^-1^; using a stock solution of 37.9 mM NaF) and of Al (10.4 μM; 281 μg L^-1^; using a stock solution of 1 mM Al in 4% HNO_3_) throughout. In order to test the effect of pH on the accumulation of Al and F, we performed the same experiment as described above, but at pH 5.5, 7.0 and 7.5 (pH adjusted with small additions of dilute HNO_3_ or NaOH). For this part of the study, we tested the one species that showed the best accumulation capacity.

Along with both treatments, a control containing algae with no added Al or F was used to monitor the regular growth of the algae in the medium. Also, a control with Al and F, but no algae, was used to monitor any abiotic changes in solution (*e.g.* losses in Al and F due to adsorption to container walls). Both controls were carried out in triplicate.

Algae were exposed during a period of four days (96 hours) in order to observe both the sorption process (short term) and the uptake process (long term). Algal growth was followed daily using a particle counter (Multisizer™ 3 Coulter Counter) and dissolved Al was measured regularly after 0, 1, 6, 24, 48, 72 and 96 h using disposable syringe filter units with encapsulated polyethersulfone membranes (0.45 μm, VWR International, model no. 28145–503). At the end of the exposure period, algal cultures were filtered using polycarbonate membranes (Millipore) with a porosity of 2 μm in order to separate algal cells from the medium and measure dissolved Al and F. Then, the cells were rinsed with a solution of ethylene diaminetetraacetic acid (EDTA; 20 μM) for a total contact time of 10 minutes. The presence of EDTA allows the desorption of Al from the algal surface and thus allow us to differentiate between [Al]_sorbed_ and [Al]_cellular_[7,9,20]. EDTA represents a suitable desorption ligand since it has a strong affinity for Al (log K_AlEDTA_ = 16.5) and it is not assimilated by algae [21,22].

Weakly sorbed F is assumed to be released by the presence of a concentration gradient when the cells are resuspended in a fluoride free medium. We were thus able to determine cell-sorbed Al and F from the filtrate. Finally, filters containing the algae were digested in a solution composed of 1 mL of concentrated nitric acid (Fisher Scientific, 70%) and 125 μL of hydrogen peroxide (Fisher Scientific, 30%). Fluoride samples were diluted with a 1:1 ratio with TISAB II (Orion 940909, Thermo Scientific), which provided a constant background ionic strength, dissociated loosely bound fluoride ions, and adjusted the solution pH. Fluoride was then measured using a fluoride ion selective electrode (Orion 9609BNWP, Thermo Scientific). Aluminium samples were acidified to 4% and kept at 4°C until analysis by ICP-AES (ion-coupled plasma atomic emission spectrometry; Vista AX, Varian).

For every step, blanks were prepared in order to determine background concentrations and to detect possible contamination. More precisely, a blank was prepared for the filtration with syringes, for the funnel filtration on the manifold, and for the digestion process. In every case, blanks were conclusive and confirmed that no measurable contamination came from the solutions, the handling, and/or the material used. Mass balances were conducted for Al in order to estimate the recovery level.

The chemical speciation of Al and F in both treatments was determined using MINEQL + (version 4.6) [23]. The software SYSTAT (version 13, Cranes Software International Ltd.) was used to compare values and locate significant differences. More precisely, after having verified the normality of the data (Kolmogorov-Smirnov test) and the homogeneity of variances (Levene’s test), a one-way ANOVA was conducted on the different values. When a significant difference was found, a post-hoc comparison test (Scheffe’s Test) was used to determine which values were responsible for those differences. In every case, the confidence interval was set to 95%.

## Conclusions

Biological methods show potential as a complement to conventional techniques to remove contaminants when concentrations are low. With this study, we can conclude that, using any of the conditions tested, F removal was not very promising. On the other hand, the removal of Al was quantified and was shown to be species-dependant. *Chlorella vulgaris* is not a good species for this type of treatment at pH 7.0 while *P. subcapitata* gave the best results with 41% of total removal and *S. obliquus* showed a sorption capacity of 13% similar to *P. subcapitata* (15%). In conclusion, *P. subcapitata* and/or *S. obliquus* could be considered depending on the expected effluent treatment time frame. The pH clearly played a role in Al accumulation. We observed better removal at neutral pH, especially at pH 7.5 where total Al removal reached 50%. Given our observation of particulate aluminium, a biotreatment in conjunction with a filtration step could result in very significant decreases in dissolved Al.

In this study, an artificial medium was used and all the conditions were controlled. It would be relevant to conduct such experiments with real effluent samples. Along with other factors, a different composition of anions and cations as well as the presence of organic matter would certainly influence the bioavailability of Al and its accumulation by algae. Since uptake mechanisms remain somewhat unknown for Al, more studies need to be undertaken. Ultimately, many more species could be tested for their potential in Al removal and especially for the removal of fluoride ions.

## Abbreviations

Al: Aluminium; CPCC: Canadian Phycological Culture Center; EDTA: Ethylene diaminetetraacetic acid; F: Fluoride; ICP-AES: Ion-coupled plasma atomic emission spectrometry; MHSM: Modified High Salt Medium; TISAB: Total ionic strength adjustment buffer.

## Competing interests

The authors declare that they have no competing interests.

## Authors’ contributions

DP conducted the experiments and wrote the initial draft of this paper as part of M.Sc. graduate programme. AB and CF co-supervised DP, contributed to design the experiments and helped to draft the manuscript. All authors read and approved the final manuscript.

## References

[B1] CCME Canadian water quality guidelines for the protection of aquatic life: Aluminium 2003 Winnipeg, Canada: Canadian Council of Ministers of the Environment

[B2] Volesky B Biosorption of heavy metals 1990 Boca Raton, FL, USA: CRC Press 396

[B3] Crist RH Martin JR Carr D Watson JR Clark HJ Crist DR Interactions of metals and protons with algae. 4. Ion exchange vs adsorption models and a reassessment of Scatchard plots; ion-exchange rates and equilibria compared with calcium alginate Environ Sci Technol 1994 28 1859 1866 10.1021/es00060a016 22175926

[B4] Crist RH Oberholser K McGarrity J Crist DR Johnson JK Brittsan JM Interaction of metals and protons with algae.3: marine algae, with emphasis on lead and aluminum Environ Sci Technol 1992 26 496 502 10.1021/es00027a007

[B5] Crist RH Oberholser K Shank N Nguyen M Nature of bonding between metallic ions and algal cell walls Environ Sci Technol 1981 15 1212 1217 10.1021/es00092a010

[B6] Wilkinson KJ Slaveykova VI Hassler CS Rossier C Physicochemical mechanisms of trace metal bioaccumulation by microorganisms Chimia 2002 56 681 684 10.2533/000942902777679957

[B7] Hassler CS Slaveykova VI Wilkinson KJ Discriminating between intra-and extracellular metals using chemical extractions Limnol Oceanogr Methods 2004 2 237 247

[B8] Stein WD Channels, Carriers, and Pumps–An Introduction to Membrane Transport 1990 San Diego, USA: Academic 326

[B9] Crémazy A Campbell PGC Fortin C The biotic ligand model can successfully predict the uptake of a trivalent ion by a unicellular alga below pH 6.50 but not above: possible role of hydroxo-species Environ Sci Technol 2013 47 2408 2415 10.1021/es3038388 23360212

[B10] Campbell PGC Fortin C Férard J-F, Blaise C Biotic Ligand Model Encyclopedia of Aquatic Ecotoxicology 2013 Netherlands, Dordrecht: Springer 237 246

[B11] Mehta SK Gaur JP Use of algae for removing heavy metal ions from wastewater: progress and prospect Crit Rev Biotechnol 2005 25 113 152 10.1080/07388550500248571 16294830

[B12] Volesky B Holan ZR Biosorption of heavy metals Biotechnol Prog 1995 11 235 250 10.1021/bp00033a001 7619394

[B13] Driscoll CT Schecher WD The chemistry of aluminum in the environment Environ Geochem Health 1990 12 28 49 10.1007/BF01734046 24202563

[B14] Côté G Étude des effets de l’aluminium sur l’algue verte Chlorella pyrenoidosa dans des milieux d’exposition contenant des fluorures. MSc Thesis 2003 Québec, Canada: Université du Québec, INRS–Eau, Terre et Environnement

[B15] Ali G Fluoride and aluminium tolerance in planktonic microalgae Fluoride 2004 37 88 95

[B16] Fortin C Campbell PGC Silver uptake by the green alga *Chlamydomonas reinhardtii* in relation to chemical speciation: influence of chloride Environ Toxicol Chem 2000 19 2769 2778 10.1002/etc.5620191123

[B17] Mohan SV Ramanaiah SV Rajkumar B Sarma PN Biosorption of fluoride from aqueous phase onto algal *Spirogyra* IO1 and evaluation of adsorption kinetics Bioresour Technol 2007 98 1006 1011 10.1016/j.biortech.2006.04.009 16762543

[B18] Bhatnagar M Bhatnagar A Jha S Interactive biosorption by microalgal biomass as a tool for fluoride removal Biotechnol Lett 2002 24 1079 1081 10.1023/A:1016086631017

[B19] Parent L Campbell PGC Aluminum bioavailability to the green alga *Chlorella pyrenoidosa* in acidified synthetic soft water Environ Toxicol Chem 1994 13 587 598 10.1897/1552-8618(1994)13[587:ABTTGA]2.0.CO;2

[B20] Schenck RC Tessier A Campbell PGC The effect of pH on iron and manganese uptake by a green alga Limnol Oceanogr 1988 33 538 550 10.4319/lo.1988.33.4.0538

[B21] Orvig C Robinson GH The aqueous coordination chemistry of aluminum Coordination chemistry of aluminum 1993 New York, NY, USA: VCH Publishers Inc 85 121

[B22] Twiss MR Errécalde O Fortin C Campbell PGC Jumarie C Denizeau F Berkelaar E Hale B Van Rees K Coupling the use of computer chemical speciation models and culture techniques in laboratory investigations of trace metal toxicity Chem Speciation Bioavailability 2001 13 9 24 10.3184/095422901782775462

[B23] Schecher WD McAvoy D MINEQL+: A Chemical Equilibrium Modeling System 2007 Hallowell, ME, USA: Environmental Research Software

